# How many steps/day are enough? for adults

**DOI:** 10.1186/1479-5868-8-79

**Published:** 2011-07-28

**Authors:** Catrine Tudor-Locke, Cora L Craig, Wendy J Brown, Stacy A Clemes, Katrien De Cocker, Billie Giles-Corti, Yoshiro Hatano, Shigeru Inoue, Sandra M Matsudo, Nanette Mutrie, Jean-Michel Oppert, David A Rowe, Michael D Schmidt, Grant M Schofield, John C Spence, Pedro J Teixeira, Mark A Tully, Steven N Blair

**Affiliations:** 1 Walking Behavior Laboratory, Pennington Biomedical Research Center, Baton Rouge, LA, USA; 2 Canadian Fitness and Lifestyle Research Institute, Ottawa, ON, Canada; 3 School of Public Health, University of Sydney, Sydney, Australia; 4 School of Human Movement Studies, University of Queensland, Brisbane, Australia; 5 School of Sport, Exercise and Health Sciences, Loughborough University, UK; 6 Department of Movement and Sports Sciences, Ghent University, Ghent, Belgium; 7 Center for the Built Environment and Health, School of Population Health, The University of Western Australia; 8 Tokyo Gakugei University, Tokyo, Japan; 9 Department of Preventive Medicine and Public Health, Tokyo Medical University, Tokyo, Japan; 10 Centro de Estudos do Laboratório de Aptidão Física de São Caetano do Sul (CELAFISCS), Brazil; 11 School of Psychological Sciences and Health, University of Strathclyde, Glasgow, UK; 12 Department of Nutrition, Pitié-Salpêtrière Hospital (AP-HP), University Pierre et Marie Curie-Paris6, Center for Human Nutrition Research Ile-de-France (CRNH-IdF), Paris, France; 13 Department of Kinesiology, University of Georgia, Athens, GA, USA; 14 Menzies Research Institute, University of Tasmania, Hobart, Australia; 15 Centre for Physical Activity and Nutrition, Auckland University of Technology, Auckland, New Zealand; 16 Faculty of Physical Education and Recreation, University of Alberta, Alberta, Canada; 17 Faculty of Human Kinetics, Technical University of Lisbon, Lisbon, Portugal; 18 UKCRC Centre for Public Health (NI), Queen's University, Belfast, Ireland; 19 Departments of Exercise Science and Epidemiology/Biostatistics, Arnold School of Public Health, University of South Carolina, Columbia, USA

## Abstract

Physical activity guidelines from around the world are typically expressed in terms of frequency, duration, and intensity parameters. Objective monitoring using pedometers and accelerometers offers a new opportunity to measure and communicate physical activity in terms of steps/day. Various step-based versions or translations of physical activity guidelines are emerging, reflecting public interest in such guidance. However, there appears to be a wide discrepancy in the exact values that are being communicated. It makes sense that step-based recommendations should be harmonious with existing evidence-based public health guidelines that recognize that "some physical activity is better than none" while maintaining a focus on time spent in moderate-to-vigorous physical activity (MVPA). Thus, the purpose of this review was to update our existing knowledge of "How many steps/day are enough?", and to inform step-based recommendations consistent with current physical activity guidelines. Normative data indicate that healthy adults typically take between 4,000 and 18,000 steps/day, and that 10,000 steps/day is reasonable for this population, although there are notable "low active populations." Interventions demonstrate incremental increases on the order of 2,000-2,500 steps/day. The results of seven different controlled studies demonstrate that there is a strong relationship between cadence and intensity. Further, despite some inter-individual variation, 100 steps/minute represents a reasonable floor value indicative of moderate intensity walking. Multiplying this cadence by 30 minutes (i.e., typical of a daily recommendation) produces a minimum of 3,000 steps that is best used as a heuristic (i.e., guiding) value, but these steps must be taken *over and above *habitual activity levels to be a true expression of free-living steps/day that also includes recommendations for minimal amounts of time in MVPA. Computed steps/day translations of time in MVPA that also include estimates of habitual activity levels equate to 7,100 to 11,000 steps/day. A direct estimate of minimal amounts of MVPA accumulated in the course of objectively monitored free-living behaviour is 7,000-8,000 steps/day. A scale that spans a wide range of incremental increases in steps/day and is congruent with public health recognition that "some physical activity is better than none," yet still incorporates step-based translations of recommended amounts of time in MVPA may be useful in research and practice. The full range of users (researchers to practitioners to the general public) of objective monitoring instruments that provide step-based outputs require good reference data and evidence-based recommendations to be able to design effective health messages congruent with public health physical activity guidelines, guide behaviour change, and ultimately measure, track, and interpret steps/day.

## Background

Around the world, physical activity guidelines are written and promoted by government and non-governmental agencies to provide direction for recommended amounts of physical activity required to benefit health, essentially answering the question "how much is enough?" These guidelines are typically expressed in terms of frequency, duration, and intensity parameters and are based on decades of epidemiological and intervention research that has almost exclusively relied on self-reported physical activity behaviours. The recent release of the U.S. Physical Activity Guidelines [1] acknowledges that "some physical activity is better than none" while maintaining a focus on time spent in moderate-to-vigorous physical activity (MVPA). The evolution of objective monitoring of physical activity using pedometer and accelerometer technology offers an opportunity to extend guidelines to include recommendations for objectively monitored parameters reflective of time spent in MVPA in the context of free-living behaviour.

The data generated by accelerometers are robust and can be downloaded and converted into time spent in various intensities of physical activity and inactivity by applying accepted accelerometer-specific cut points (i.e., intensity-linked activity counts that represent a proprietary digitized integration of a movement event and its acceleration). Although the importance of these data in terms of studying frequency and duration of intensity-specific activity is unquestionable, accelerometers typically are relatively expensive and require additional personnel time and expertise to manage and manipulate the data to derive these end points. In contrast, pedometers are inexpensive, easy to use, and the step output is readily available (i.e., digitally displayed on screen) and easily interpretable as an indicator of overall volume of physical activity. The output of pedometers and accelerometers is clearly related [2]. Although accelerometers are now also being used to capture and describe step data in nationally representative surveys [3], pedometers are more likely to be used in public health and clinical applications and adopted by the general public due to their relative low cost, practicality, and interpretability.

The various and emerging step-based recommendations from around the world are catalogued in Table 1 and reflect public interest in such guidance. As can be seen from the table, there appears to be a wide discrepancy in the range of step-based recommendations that are being communicated. Yet internationally, similar frequency-, duration-, and intensity-based public health guidelines are endorsed: 30 minutes (at times up to 60 minutes) per day (or 150-210 minutes/week) in MVPA, typically in minimal 10 minute bouts [4-9]. These widely accepted, evidence-based adult public health physical activity guidelines were originally formulated in terms of preventing morbidity and mortality. As framed, these frequency-, duration-, and intensity- based guidelines imply that the recommended dose of physical activity should be taken *over and above *a baseline level (of lower intensity activities) that has never been explicitly described, and may in fact be changing as a result of societal trends, which further complicates the issue. Public health guidelines [1] also now state that, especially for inactive adults, "some physical activity is better than none," and this recognition sets the stage for an expanded yet still compatible step-based message that also accommodates recommended amounts of time in MVPA.

**Table 1:** Government/agency/professional organization step-based recommendations from around the world

Government/ agency/ professional organization	Step-based recommendation
Queensland Health (Australia)	Sponsors 10,000 Steps: "aims to increase the day-to-day activity of Australians by encouraging you to use a step-counting pedometer to accumulate 'incidental' physical activity as part of your everyday living" (http://www.10000steps.org.au/)

National Heart Association of Australia	Produced a brochure in 2009 "Making every step count" ISBN 978-1-921226-71-7, http://www.heartfoundation.org.au, that says "a suggested target for healthy adults is 10,000 steps per day."

U.S. President's Challenge Physical Activity and Fitness Awards Program	Recommends 8,500 steps/day for adults, and 13,000 and 11,000 steps/day for boys and girls respectively (http://www.presidentschallenge.org/challenge/active/index.shtml)

America on the Move	Promotes walking an extra 2,000 steps in addition to eating 100 less calories each day to stop weight gain(http://aom3.americaonthemove.org/)

National Obesity Forum (U.K)	Indicates that 3,000 to 6,000 steps/day is sedentary, 7,000 to 10,000 steps is moderately active, and > 11,000 steps/day is very active. (http://www.nationalobesityforum.org.uk/healthcare-professionals-mainmenu-155/treatment-mainmenu-169/192-useful-tools-and-agencies.html)

Northern Ireland's Public Health Agency	Promotes an additional 30 minutes of daily walking or 3000 steps (http://www.getalifegetactive.com/adults/walking/walking)

Ministry of Health, Labour and Welfare of Japan [77]	Recommends: "for individuals who intend to promote health mainly through physical activity, a daily walk of 8,000 to 10,000 steps is set as the target. The report indicates that 8,000 to 10,000 steps/day is approximately equivalent to 60 minutes of walking per day at an intensity of 3 METs, and that it is also approximately equivalent to 23 MET-hours/week of MVPA which is the recommended physical activity level in this guideline.

In order to avoid being construed as simply another source of confusion and disagreement, it makes sense that any step-based recommendation should be harmonious with existing physical activity guidelines. They are "not intended to supplant existing public health recommendations, but rather supplement them" [10]. However, there is an opportunity to posit a total number of steps/day so that both habitual activity levels (taken in the course of free-living and not necessarily of at least moderate intensity) and suggested increments in physical activity that meet frequency-, duration-, and intensity-based parameters are considered in the recommended 'dose.' The question "How many steps/day are enough?" has been previously reviewed [11,12]. The literature related to objective monitoring of physical activity is growing at a considerable rate and it is again time to address this question. The purpose of this review therefore was to update and identify gaps in the evidence to inform step-based recommendations congruent with current physical activity guidelines and otherwise to extend guidelines to include recommendations for "How many steps/day are enough?"

## Methods

In February 2010, the Public Health Agency of Canada (PHAC) commissioned a literature review designed to identify how many steps are approximately equivalent to public health guidelines in children/adolescents, adults, and older adults/special populations. A professional librarian identified 1,594 articles by conducting a search of English language literature published since 2000 in CINAHL, ERIC, MEDLINE, PsycINFO, SocINDEX, and SPORTDiscus using the keywords (pedomet* or acceleromet*) and step* and ((physical activity) or walk*). This list was reduced to 837 articles once duplicates, remaining non-English language articles, dissertations, non-peer reviewed articles, and those obviously not dealing with step-defined human physical activity were removed. Abstracts for this reduced list of articles were initially read by the first author to identify relevant articles and electronic copies of these were assembled using Endnote X3 (Thomson Reuters, New York). Additional articles were identified from article reference lists. Relevant content was tabulated and/or summarized by the first author. Select researchers from around the world with experience in collecting objectively monitored step data were invited to identify any missing literature (including known in-press articles), critically review the report, edit check and verify assembled data, and intellectually contribute by participating in the writing of a series of consensus documents (children/adolescents [13], adults, and older adults/special populations [14]) intended to provide step-based recommendations congruent with public health guidelines (given the limitations detailed below). This specific review is focused on healthy adults approximately 20-65 years of age, although the upper limit was not rigid (as driven by the identified literature), and living without disability or chronic illness. The child/adolescent [13] and older adult/special populations [14] literature is reviewed separately. No other inclusion criteria were used other than relevance to the question at hand.

Identified themes emerged as the literature was reviewed and provide a structure for the remainder of this article: 1) normative data (i.e., expected values); 2) incremental changes expected from interventions; 3) controlled studies that determine exact step-based conversions of timed behaviour; 4) computing a step translation of duration- and intensity-based physical activity guidelines (e.g., steps/day associated with time spent in MVPA); 5) directly measured steps/day indicative of minimal time in MVPA taken under free-living conditions; and, 6) steps/day associated with various health outcomes. Essentially, each section represents a 'mini-review.' At times the search strategy was exhaustive and the exact number of articles identified is presented under the appropriate heading below (e.g., controlled studies). Exceptions occur in the case of identified current review articles (e.g., normative data, interventions). The findings of these reviews were simply summarized herein and select original articles are referred to only to make specific points. Where appropriate, details of studies are presented in tables; inconsistencies in reporting within and across tables (e.g., instrument brand, model, etc.) reflect underlying reporting inconsistencies between original articles.

## Results

### Normative data (expected values)

An early review of 32 studies published between 1980 and 2000 [15] indicated that healthy younger adults (approximately 20-50 years of age) take 7,000-13,000 steps/day. Many more studies of step-defined physical activity measured using pedometers and accelerometers are published today, including a more recent review article of adult normative data. Specifically, Bohannon [16] used a meta-analytic approach to summarize and present steps/day taken by healthy adults (18+ years of age). Forty-two studies published between 1983 and 2004 were identified. Reported values for adults under 65 years of age ranged from approximately 5,400 steps/day (in a U.S. sample of multiethnic women mean age 54.2 years [17]) to 18,000 steps/day (in a sample of Amish men mean age 34 years [18]). Excluding the Amish sample, overall mean steps/day was 9,448 (95% CI = 8,899-9,996). The NHANES accelerometer data were adjusted to facilitate interpretation on a pedometer-based scale, since accelerometers typically detect more steps than pedometers [19,20]. The findings indicate that, on average, U.S. adults take approximately 6,500 steps/day [3], not too different from two other U.S. estimates based on pedometer data: Colorado (≅6,800 steps/day) [21] and South Carolina (≅5,900 steps/day) [22]. A more recent article reported that U.S. adults average approximately 5,100 steps/day when measured by a pedometer [23]. In contrast, other representative samples indicate that Japanese people aged 15+ years take an average of approximately 7,200 steps/day [24], Western Australians aged 18+ years take approximately 9,600 steps/day [25], Belgian adults aged 25-75 years take approximately 9,600 steps/day [26], and Swiss adults aged 25-74 years of age take approximately 8,900 steps/day (women) and 10,400 steps/day (men) [27]. Despite differences in instrumentation used, the ability to compare results across studies that have used research-quality pedometers is reasonably good [28].

In 2004 Tudor-Locke and Bassett [11] introduced the concept of a graduated step index for healthy adults: 1) < 5,000 steps/day ('sedentary'); 2) 5,000-7,499 steps/day ('low active'); 3) 7,500-9,999 steps/day ('somewhat active'); 4) ≥10,000-12,499 steps/day ('active'); and 5) ≥12,500 steps/day ('highly active'). This index was revisited and given additional support in 2008 as part of an updated review of "How many steps/day are enough?" [12] and in 2009 the original 'sedentary' level (i.e., < 5,000 steps/day) was further split into two additional graduations: < 2,500 steps/day ('basal activity') and 2,500-4,999 steps/day ('limited activity') [3]. The utility of this graduated step index has been assessed in terms of discriminating individuals by body mass index (BMI) [29] and reflecting increased cardiometabolic risk [30] (reviewed in more detail below). Thus, step-based estimates of U.S. adults' habitual physical activity would classify the population as 'low active' according to this existing step-defined physical activity scale [11,12].

Sixteen free-living healthy adult studies (Table 2) were identified that reported the percentage of their samples achieving specified step-defined cut points, including applying cut points associated with the graduated step index described above. Five used 10,000 steps/day as an exclusive cut point (no other cut point was considered). Eight reported using the graduated step index originally proposed by Tudor-Locke and Bassett [11]. Two studies of South African samples that also made use of the graduated step index were excluded from Table 1 because their lower age limits extended into adolescence [31,32], beyond the scope of this specific review. Apparent patterns from Table 1 include: younger adults are more likely to achieve 10,000 steps/day, U.S. samples are more likely to take < 5,000 steps/day compared to Australian samples, and those with lower incomes are also more likely to take < 5,000 steps/day than high income earners. The studies that have reported data using versions of the graduated step index provide more robust (i.e., more levels) data for comparison and tracking purposes than those that have only reported relative attainment of any single value of steps/day.

**Table 2:** Studies of free-living behaviour reporting percent of participants meeting select step-defined cut points in adults

First Author	Sample Characteristics	Instrument	Monitoring Frame	Steps/day cut points used	% Meeting Specified Cut point
Tudor-Locke [22] USA 2004	76 men, 133 women; population-based survey of Sumter County, South Carolina; 18+ years of age	Yamax SW-200, Yamax Corporation, Tokyo, Japan	7 days	5,000 9,000 10,000	44% < 5,000 19.6 ≥ 9,000 13.9% ≥ 10,000

Miller [50] Australia 2004	74 men, 111 women; workplace employees; 18 to 75 years	Yamax SW 700	7 days	10,000	Men: 24.4% Women: 34.2%

Behrens [51] USA 2005	18 men, 18 women; college students; 23.3 ± 3.1 years	Digi-walker (Model DW-200, Yamax, Tokyo, Japan) Actigraph 7164, Manufacturing Technology Incorporated, Fort Walton Beach, FL	7 days	10,000	80%

Wyatt [21] USA 2005	344 men, 386 women; 18+ years of age; Colorado statewide representative sample	Yamax SW-200, Yamasa Corporation, Tokyo, Japan	4 days	Adult Graduated Step Index	33% < 5,000 29% 5,000-7,499 22% 7,500-9,999 9% 10,000-12,500 7% > 12,5000

Behrens [78] USA 2005	204 men, 237 women; college students; 20.05 ± 1.82 years	Actigraph 7164, Manufacturing Technology Incorporated, Fort Walton Beach, FL	7 days	10,000	Overall: 67.35% Men: 69.6% Women: 65.4%

Hornbuckle [79] USA 2005	69 women; self-identified African American volunteers; 40-62 years of age	New Lifestyles Digi-Walker SW-200, New Lifestyles, Inc., Lees Summit MO	7 days	Adult Graduated Step Index	38% < 5,000 46% 5,000-7,499 16% ≥7,500

Bennett [80] USA 2006	153 men, 280 women; Multiethnic low-income housing residents; 18 to 70+ years	Yamax SW200	5 days	sedentary index: 5,000; normative for healthy adults: 7,000-13,000; normative for healthy older adults: 6,000-8,500	56% < 5,000 24% 7,000-13,000 8% of those 50+ took between 6,000 and 8,500

McCormack [25] Australia 2006	205 men, 223 women; state wide community sample; ≥18 years	Yamax Digi-walker SW-700	7 days	10,000	Men: 50.2% Women: 40.8%

De Cocker [26] Belgium 2007	598 men, 624 women; random sample from public record office; 25 to 75 years	Yamax Digiwalker SW-200 (Yamax, Tokyo, Japan)	7 days	Adult Graduated Step Index	12.9% < 5000 19.4% 5000-7499 26.2% 7500-9999 21.1% 10,000-12,500 20.5% > 12,5000

De Cocker [81] Belgium 2008	146 men, 164 women; healthy adults; 38.7 ± 11.9 years	Yamax Digiwalker SW-200, (Yamax, Tokyo, Japan)	7 days	7,500 10,000 12,500	≥7,500: 80.6% ≥10,000: 45% ≥12,500: 39.4%

Mitsui [82] Japan 2008	62 men,117 women; recruited through medical check-up at public health center; 48 to 69 years	EM-180, YAMASA, Tokyo, Japan	7 days	Adult Graduated Step Index	Men 30.6% < 5000 25.8% 5000-7499 17.7% 7500-9999 25.8% ≥10000 Women 28.2% < 5000 35.0% 5000-7499 24.8% 7500-9999 12.0% ≥10000
Payn [74] USA 2008	25 men, 60 women; community sample, ambulatory and without cognitive impairment; 45+ years	Yamax Digi Walker SW-200, Yamax USA, Inc., San Antonio, TX	7 days	Adult Graduated Step Index	29.4% ≤ 5000 43.5% 5001-9999 27.1% ≥ 10,000

McKercher [59] Australia 2009	766 men, 869 women; young adults participating in a longitudinal study; 26 to 36 years	Yamax Digiwalker SW-200	7 days	Adult Graduated Step Index	Men 8.2% < 5,000 29.6% 5,000-7,499 27.7% 7,500-9,999 19.7% 10,000-12,499 14.8% 12,500+ Women 6.7% < 5,000 28.2% 5,000-7,499 33.5% 7,500-9,999 21.1% 10,000-12,499 10.6% 12,500+

Schmidt [30] Australia 2009	887 men, 906 women; 26 to 36 years	Yamax SW-200	7 days	Adult Graduated Step Index	Men 7.8% 0-4,999 27.9% 5,000-7,499 27.3% 7,500-9,999 21.4% 10,000-12,999 15.7% 12,500+ Women 6.2% 0-4,999 27.9% 5,000-7,499 33.2% 7,500-9,999 21.3% 10,000-12,999 11.4% 12,500+

Tudor-Locke [83] USA 2011	1781 men, 1963 women; NHANES participants (nationally representative); 20 to 85+ years	ActiGraph AM-7164; censored data to approximate pedometer outputs	7 days	Adult Graduated Step Index with additional sedentary categories	Men 14.1% < 2,500 20.6% 2,500-4,999 24.2% 5,000-7,499 19.3% 7,500-9,999 10.9% 10,000-12,499 10.8% 12,500+ Women 14.1% < 2,500 20.6% 2,500-4,999 24.2% 5,000-7,499 19.3% 7,500-9,999 13.2% 10,000-12,499 10.8% 12,500+

Clemes [84] UK 2011	44 men 52 women; 18 to 65 years	SW-200 pedometer (New Lifestyles, Inc., Lees Summit, MO)	4 weeks in summer and again in winter	10,000 steps/day	Normal weight Summer 60% ≥ 10,000 Winter 35%≥ 10,000 Overweight Summer 43%≥ 10,000 Winter 35%≥ 10,000

Adult Graduated Step Index [11]: 1) < 5,000 steps/day ('sedentary'); 2) 5,000-7,499 steps/day ('low active'); 3) 7,500-9,999 steps/day ('somewhat active'); 4) ≥10,000-12,499 steps/day ('active'); and 5) ≥12,500 steps/day ('highly active'). These categories were reinforced in an updated review in 2008 [12] and in 2009 the original 'sedentary' level was segmented into two additional levels: < 2,500 steps/day ('basal activity') and 2,500 to 4,999 steps/day ('limited activity') [3].

### Interventions

Three different meta-analytic reviews of controlled and/or quasi-experimental studies have summarized the effects of pedometer-based physical activity interventions in adults, published in 2007 [33], 2008 [34], and 2009 [35], respectively. In addition, a selective review [36] has re-examined the studies published in the two earlier reviews [33,34] to gain insight into why pedometers are effective behaviour change instruments. We therefore only offer a brief summary of these findings here. The use of pedometers in behaviour modification programs increases physical activity by approximately 2,000 [35] to 2,500 steps/day [33,34]. This level of increase is associated with modest weight loss [33,34] and improvements in blood pressure [33]. Studies employing a step goal [33], and in particular a 10,000 steps/day goal [35], appear to have had the greatest impact on increasing physical activity. As previously noted, however [36], few studies have evaluated alternative goals to 10,000 steps/day, and no study to date has systematically evaluated dose-response effects of different steps/day goals. Therefore it may be premature to make firm conclusions about the efficacy, effectiveness, or appropriateness of any specific step-based goal in terms of behaviour change. It is possible that working towards *any *goal that represents an increase over baseline values is likely to be much more important, from a behavioural perspective at least, than the value of the exact target number [36]. It is important to acknowledge that the nature of a goal (i.e., an objective that defines intention at the level of the individual) differs from, but may overlap, the concept of step-based recommendations consistent with public health physical activity guidelines pursued herein. It is also clear that other cognitive and behavioural strategies are important to incorporate into successful intervention programs [37].

### Controlled studies

Eight controlled studies (Table 3) have been conducted using treadmills [38-43], tracks [40], or hallways [44] to determine exact step-based conversions of timed continuous ambulation. Sufficient data were reported in all these studies to summarize cadence (steps/minute values), speed (reported in either miles/hr or km/hr, otherwise converted here), and METs as reported, imputed, or otherwise inferred from Compendium of Physical Activity [45] values and summarized in Table 4. Each of these strategies is indicated in the table notes. The correlation between the mean values for steps/minute and speed (miles/hr or km/hr) is presented in Table 4 is *r *= 0.97 (strong). The correlation between steps/minute and MET level is also strong (*r *= 0.94). Cadence is known to be the primary strategy for increasing free-living walking speed [46] and although stride lengthening becomes relatively more important in running, cadence still increases with running speed [47]. The five studies that directly measured the number of steps and verified absolutely-defined moderate intensity activity [38-40,43,44] came to similar conclusions: despite inter-individual variation, 100 steps/minute represents a reasonable heuristic (i.e., guiding) value for absolutely-defined moderate intensity walking.

**Table 3:** Controlled study designs that have informed "how many steps/day are enough?" in adults

Reference	Sample Characteristics	Step Counting Instrumentation	Protocol	Analysis strategy	Findings
Welk [41] 2000 USA	17 males, 14 females Cooper Aerobics Center employees 29.0 ± 8.0 years	Yamax Digi-Walker (Yamax Inc., Tokyo, Japan), observed tally	walk/jog a track and/or treadmill mile at 4, 6, and 7.5 miles/hr (6.4, 9.66, and 12.8 km/hr*)	steps taken for each pace extrapolated from 4mph pace steps in 30 minutes moderate intensity	3,800-4,000 steps would approximate 30 minutes of moderate intensity walking

Tudor-Locke [38] 2005 USA	25 males, 25 females university community 18 to 39 years	Yamax SW-200, Yamax Corp., Tokyo, observed tally	6-minute treadmill bouts at 4.8, 6.4, and 9.7 km/hr	V0_2 _from expired gases Regression METs predicted from steps/minute	3,000-4,000 steps in 30 minutes of moderate intensity walking based on a threshold cadence of 100 steps/min

Marshall [39] 2009 USA	39 males, 58 females community sample of Latino adults 32.1 ± 10.6 years	Yamax SW-200, observed tally	6-minute treadmill bouts at 2.4, 3.0,3.5, 4.1 miles/hr (3.86, 4.83, 5.64, and 8.04 km/hr*)	V0_2 _from expired gases; METs predicted from steps/minute multiple regression, mixed modelling, receiver operating curves	Inter-individual variation apparent however, minimally 3,000 steps in 30 minutes of moderate intensity walking based on a threshold cadence of 100 steps/min

MacPherson [42] 2009 New Zealand	12 males, 15 females university students 18 to 39 years	Observed tally	10,000 steps on treadmill at 3.2 and 6.4 km/hour	time to complete and PAEE kcal from Tritrac-R3D accelerometer	most participants could achieve at least 150 kcal in energy expenditure with 10,000 steps at the slow walk (median 255 kcal, range 148-401). Faster walking produced a higher energy expenditure (median 388 kcal, range 294-901).

Beets [44] 2010 USA	9 males, 11 females; healthy adults; 26.4 ± 4.5 years	Observed tally	6-minute hallway bouts at 1.8, 2.7, 3.6, 4.5, and 5.4 km/hr*	Random effects models to predict steps/min from METs and anthropometric measures	Inter-individual variation apparent however, minimally 3,000 steps in 30 minutes of moderate intensity walking based on a threshold cadence of 100 steps/min

Rowe [40] 2011 UK, USA	37 males, 38 females; university students, employees, and their families; 32.9 ± 12.4 years	Observed tally	6-minute treadmill bouts at randomly assigned sets of slow (mean 4.3 km/hr), medium (5.0 km/hr), fast (5.8 km/hr) speeds And Over-ground track walks (at least 4 minutes) at treadmill-determined cadences (cued by metronome)	Mixed model regression analysis to predict METs from cadence, anthropometric measures, stride length	Inter-individual variation apparent however, minimally 3,000 steps in 30 minutes of moderate intensity walking based on a threshold cadence of 100 steps/min

Abel [43] 2011 USA	9 males, 10 females; university population, frequent runners; 28.8 ± 6.8 years	Observed tally	10-minute treadmill bouts at walking (3.24, 4.8, and 6.42 km/hr*) and running speeds (8.04, 9.66, 11.28 km/hr*)	Linear and non-linear regression analysis to predict METs from cadence Mixed ANOVA: Between subjects (sex), within subject effect of speed for cadence, stride length, VO_2_, and METs	Inter-individual variation apparent however, 100 steps/minute a reasonable estimate of moderate intensity walking

*reported speeds converted to km/hr.

**Table 4:** Speed, MET levels, and cadence from track, treadmill, and hallway walking/running studies of adults

Reference	Speed (miles/hr)	Speed (km/hr)	MET	Cadence (spm)
Beets [44]	1.12^A^	1.8^A^	2.0^B^	64^C^

Beets [44]	1.68^A^	2.70^A^	2.4^B^	81^C^

MacPherson [42]	1.99^D^	3.2	2^E^	93

Abel [43]	2.01^F^	3.24^F^	3.1^B^	96^C^

Beets [44]	2.24^A^	3.6^A^	2.7^B^	96^C^

Marshall [39]	2.4	3.86^G^	3.09^H^	109^I^

Rowe [40]	2.7	4.3	2.94^J^	102

Beets [44]	2.8^A^	4.50^A^	3.2^B^	106^C^

Abel [43]	2.98^F^	4.80^F^	4.0^B^	114^C^

Tudor-Locke [38]	2.98^D^	4.8	3.60	108^C^

Marshall [39]	3	4.83^G^	3.73^H^	115^I^

Rowe [40]	3.1	5.0	3.46^J^	114

Beets [44]	3.36^A^	5.40^A^	3.9^B^	115^C^

Marshall [39]	3.5	5.64^G^	4.94^H^	124^I^

Rowe [40]	3.6	5.8	4.2^J^	125

Abel [43]	3.99^F^	6.42^F^	5.5^B^	127^C^

Tudor-Locke [38]	3.98^D^	6.4	5.25	127^C^

Welk [41]	4	6.44	5.25^K^	129^L^

MacPherson [42]	3.98^D^	6.4	5.25^K^	129

Marshall [39]	4.1	6.60^G^	6.85^H^	134^I^

Abel [43]*	5.0^F^	8.04^F^	9.18^B^	158^C^

Abel [43]*	6.0^F^	9.66^F^	10.93^B^	165^C^

Welk [41]*	6	9.66	10^M^	163^N^

Tudor-Locke [38]*	6.02^D^	9.7	10.00	161^C^

Abel [43]*	7.01^F^	11.28^F^	12.98^B^	170^C^

Welk [41]*	7.5	12.08	12.5^O^	165^P^

* Jogging/running

Note: Superscripts denote values derived from information contained in original manuscript

^A ^Converted from reported meters/second

^B ^METs determined by weighted average METs reported for males and females

^C ^Cadence determined by weighted average spm reported for males and females

^D ^Converted from reported km/hr

^E ^Compendium code 1179: walking on job, less than 2.0 mph (in office or lab area), very slow

^F^Converted from reported meters/minute

^G ^Converted from reported miles/hr

^H ^METs determined by weighted average METs for normal weight, overweight, obese

^I ^Cadence determined by weighted average hand-counted spm for normal weight, overweight, obese

^J ^Converted from reported VO_2_

^K^MET assumed to be the same as that for 6.4 km/hr pace in Tudor-Locke et al. [38]

^L^Cadence determined by dividing weighted mean steps for men and women (1936) by time taken to complete a mile (15 min)

^M^Compendium code 12050: running, 6 mph (10 minute mile)

^N^Cadence determined by dividing weighted mean steps for men and women (1631) by time taken to complete a mile (10 min)

^O^Compendium code 12080: running, 7.5 mph (8 minute mile)

^P^Cadence determined by dividing weighted mean steps for men and women (1317) by time taken to complete a mile (8 min)

#### Computed step count translations for physical activity guidelines

As noted above, five separate studies can be used to support the assertion that 3,000 steps in 30 minutes is approximately equivalent to at least moderate intensity walking in adults, based on a cadence of 100 steps/minute [38-40,43,44]. To be considered a true translation of public health guidelines' focus on time in MVPA, however, these steps should be of at least moderate intensity (i.e., be ≥100 steps/minute), accumulated in at least 10 minute bouts, and should be taken *over and above *some baseline level of steps/day indicative of sedentarism. Since a value of ≤5,000 steps/day had been proposed as a 'sedentary lifestyle index' [11,12,48], summing this value and the supplemental steps/day considered minimally representative of recommended amounts of time in MVPA produces a floor value of approximately 8,000 steps/day. Some physical activity guidelines recommend up to 60 minutes of activity that is of at least moderate intensity [6,9]. Multiplying 60 minutes by 100 steps/minute results in 6,000 steps, that when added to a 'sedentary' level of 5,000 steps/day produces a total value of 11,000 steps/day. Therefore, a simple arithmetical translation of free-living physical activity that also includes recommended amounts of time in MVPA is 8,000 to 11,000 steps/day for adults, applied with the caveats listed above, and if expressed as a daily recommendation.

It is important to emphasize that these calculations consider only activities that generate steps. There are, of course, a wide range of human activities that may or may not generate steps, for example, those that may include upper body movement. However, bipedal locomotor activity is a fundamental aspect of human movement. Additionally, it has been shown that wrist-worn accelerometers add little extra information to those worn at the waist (and therefore are also most sensitive to ambulatory activity detected while on the wrist) [49]. The calculation above focused on adding recommended amounts of MVPA to baseline physical activity levels and therefore presumes 30 minutes of MVPA in a day. Some public health guidelines now clearly promote 150 minutes/week as the minimal amount of health-related moderate intensity [1,7]. A computed translation of this expression is 15,000 steps/week, again based on the 100 steps/minute heuristic value described above. Considering 7 days at a baseline level of 5,000 steps/day (or 35,000 steps/week), adding these extra 15,000 steps/week (for a total of 50,000 steps/week), and averaging over 7 days, produces an average of approximately 7,100 steps/day. Adding an extra 30,000 steps/week (i.e., up to 300 minutes/week [1,7]), produces an overall estimate of approximately 9,300 steps/day averaged over a week.

In summary, a computed translation of daily free-living ambulatory physical activity for adults that includes allowance for recommended amounts of time in MVPA is 8,000 to 11,000 steps/day. Allowing for a more flexible accumulation pattern that may include some "off" days, and averaged across a week, the estimate is 7,100 to 9,300 step/day. Together these estimates span 7,100 to 11,000 steps/day. In both cases, it remains important to emphasize that at least a portion of these steps (3,000 for the daily accumulation and 15,000 of the weekly total accumulation) are minimally taken at an intensity of at least 100 steps/minute (i.e., moderate intensity, absolutely defined), and in bouts of at least 10 minutes.

### Direct studies of step equivalents of physical activity guidelines

Six studies (Table 5) were identified that have attempted to provide steps/day translations of recommended amounts of either time spent in MVPA or energy expended (kcal) in healthy adults. Tudor-Locke et al. [48] reported that people who averaged 30 minutes/day of accelerometer-determined MVPA also accumulated 8,000 pedometer-determined steps/day when the two instruments were worn concurrently. Miller and Brown [50] reported that working adults who self-reported accumulating at least 150 minutes of MVPA in a week averaged 9,547 steps/day. Behrens et al. [51] reported that college students who accumulated at least 30 minutes of moderate intensity activity (vigorous intensity not considered) averaged 11,822 steps/day. In the latter two studies, mean values of the sample can be influenced by skewed data, and the process does not effectively capture a threshold value necessarily associated with achieving public health guidelines.

**Table 5:** Studies that have attempted to set steps/day cut points in adults relative to time spent in MVPA or energy expended

First Author	Sample Characteristics	Instrument	Monitoring Frame	Analytical Strategy	Findings
Tudor-Locke [48] 2002 USA	27 men, 25 women university community 38.2 ± 12.0 years	Yamax SW-200, Yamax Corporation, Tokyo, Japan; CSA 7164 Version 2.2, Computer Science Applications, Inc., Shalimar, FL	7 days	Mean steps/day associated with the step/day quartile distribution in which participants accumulated an average of 30 min/day accelerometer-determined MVPA	8,000 steps/day corresponded with accumulating 30 minutes of MVPA people taking > 12,500 took more moderate and vigorous activity than any other group

Miller [50] 2004 Australia	74 men, 111 women workplace employees 18 to 75 years	Yamax SW 700; Active Australia questionnaire	7 days	Steps/day equivalent to 150+ minutes/week self-reported MVPA	Those who met guidelines averaged 9,547 ± 2,655 steps/day

Behrens [51] 2005 USA	18 men, 18 women college students 23.3 ± 3.1 years	Digi-walker (Model DW-200, Yamax, Tokyo, Japan) Actigraph 7164, Manufacturing Technology Incorporated, Fort Walton Beach, FL	7 days	Steps/day related to 30+ minutes of accelerometer-determined moderate physical activity	11,822 steps/day

Jordan [52] 2005 USA	111 postmenopausal women intervention participants 45-75 years	Accusplit Eagle 120 (AE 120)	7 days	Steps/day associated with attaining prescribed and verified exercise equivalent to 120-150 min/week or 8kcal/kg/week EE	3-4 days of 10,000 steps/day met energy expenditure guidelines for the week or approximately 7300 steps/day (imputed from reported data)

Macfarlane [53] 2008 China	30 men, 19 women apparently healthy 15 to 55 years	SW-700, Yamax Corporation., Tokyo, Japan MTI 7164, MTI Actigraph, Fort Walton Beach, FL Tritrac RT3, Stayhealthy INC., Monrovia, CA Heart rate monitor, Team system, Polar OY, Finland	7 days	Selected 25th percentile of steps/day distribution; examined sensitivity/specificity of achieving 30 minutes MVPA measured by various instruments	8,000 steps/day

Tudor-Locke [83] 2011 USA	1781 men, 1963 women; NHANES participants (nationally representative); 20 to 85+ years	ActiGraph AM-7164; censored data to approximate pedometer outputs	7 days	Step-defined activity category where at least 30 minutes of MVPA was accumulated	Men who took 7,500-9,999 steps/day accumulated 38 minutes MVPA; women who achieved 10,000-12,499 steps/day accumulated 36 minutes of MVPA (women who achieved 7,500-9,999 steps/day accumulated 25 minutes of MVPA

Jordan et al. [52] described total steps/day associated with attaining prescribed and verified exercise equivalent to 120-150 minutes/week or 8 kcal/kg/week of energy expenditure in a sample of post-menopausal women participating in an intervention study. They found that 3-4 days of 10,000 steps/day met energy expenditure guidelines for the week, and when considered along with data collected beyond the formal exercise setting, that is, in the course of daily living outside of exercise sessions and on non-exercise days, was equivalent to approximately 7,300 steps/day (imputed from data reported in the original article). MacFarlane et al. [53] selected the 25th percentile of steps/day distribution in 49 Hong Kong Chinese people aged 15-55 years, examined sensitivity/specificity of achieving 30 minutes MVPA measured by various instruments across quartiles of steps/day distribution, and reported that the 25^th ^percentile value of 8,000 steps/day provided the best overall accuracy, sensitivity and specificity compared with higher quartile splits.

Finally, Tudor-Locke et al. [54] adjusted the 2005-2006 NHANES accelerometer data to more closely represent pedometer-based scaling and considered concurrently detected minute-by-minute step and activity count data from over 3,500 individuals with at least one valid day of wear time defined as 10/24 hours/day. Considering any minute spent in MVPA, they reported that 30 minutes/day was associated with approximately 8,000 steps/day for both men and women. A focused analysis on a subsample of participants with 7 valid days indicated that 150 minutes/week of MVPA was associated with approximately 7,000 steps/day (or 49,000 steps/week). The authors concluded that 7,000 to 8,000 steps/day, acknowledging that more is better, is a reasonably simple message that is also congruent with public health recommendations focused on minimal amounts of MVPA. A caveat is that these data considered any minute above MVPA, and therefore do not reflect an exact translation of public health guidelines that include a directive for minimal bout lengths. However, the chasm between these guidelines that have been traditionally based on self-reported activity and objectively monitored activity has been pointed out previously by users of these NHANES data [55].

In summary, directly studied estimates of free-living behaviour suggest that a total daily volume of ambulatory physical activity associated with meeting minimal amounts of MVPA is at least 7,000-8,000 steps/day. This range is similar to the threshold produced from the assumption-based computations above (i.e., 7,100 steps/day). Collectively, the results suggest that the designation of 'active' originally reserved for achieving at least 10,000 steps/day [11,12], actually encompasses a range that begins as low as 7,000 steps/day if 'active' is intended to indicate likelihood of achieving recommended amounts of weekly MVPA. Spread out over a week, more modest increases of ≅ 2,800 steps on three days/week, in line with just 50% of public health guidelines, and relative to a sedentary baseline (i.e., ≅ 4,700 steps/day) have produced important improvements in a number of health outcomes [52,56-58]. This is in keeping with the recent physical activity guidelines [1] that acknowledge that, especially for inactive adults, "some physical activity is better than none."

### Steps/day associated with various health outcomes

Although this section does not deal directly with a step-based translation of existing physical activity guidelines, five cross-sectional studies were identified that have attempted to set steps/day cut points relative to any health-related outcome, and these fit under the general purpose of this review to consider "how many steps/day are enough?" McKercher et al. [59] reported that women who achieved ≥ 7,500 steps/day had a 50% lower prevalence of depression than women taking < 5,000 steps/day. No additional benefit for depression was observed from attaining higher step-defined physical activity levels. Men who achieved ≥ 12,500 steps/day also had a 50% reduction in prevalence of depression compared with those taking < 5,000 steps/day. Only the women's results were statistically significant.

Krumm et al. [29] examined the relationship between pedometer-determined steps/day and body composition variables in 93 post-menopausal women. In relation to BMI, a linear relationship was observed such that women who took 5,000-7,500 steps/day had a significantly lower BMI than those who took < 5,000 steps/day. Further, women who took 7,500-9,999 steps/day had a significantly lower BMI than those who took 5,000-7,500 steps/day. There was no significant difference in BMI between women who took 7,500-9,999 steps/day and those who took > 10,000 steps/day.

Although Dwyer et al. [60] did not expressly set any specific steps/day cut point, they did document an inverse cross-sectional relationship between steps/day and markers of obesity in a population-based adult sample. Further, the logarithmic nature of the relationship was such that greater relative differences in waist circumference and BMI were observed for those taking habitually lower steps/day. Specifically, an extra 2,000 steps/day for someone habitually taking only 2,000 steps/day was associated with a 2.8 cm lower waist circumference in men compared with 0.7 cm lower for men already walking 10,000 steps/day. The corresponding values for potential reductions in waist circumference for women were 2.2 and 0.6 cm, respectively, for a 2,000 step addition to the two habitual walking level examples. Not surprisingly, there were larger differences in both waist circumference and BMI between those reporting 2,000 steps/day and those reporting higher counts of 10,000, 15,000 or 20,000 steps/day, but the relative benefits of small differences at lower habitual levels were still notable.

Tudor-Locke et al. [61] applied a contrasting groups method to identify optimal steps/day related to BMI- defined normal weight vs. overweight/obese in an amalgamated data base featuring pedometer and BMI data that were independently collected but using similar protocols and the same type of pedometer from Australia, Canada, France, Sweden, and the USA. Despite data limitations (e.g., fewer data available for men than women), the researchers suggested that a total number of steps/day related to a normal BMI in adults would range from 11,000 to 12,000 in men and from 8,000 to 12,000 in women, and that values were consistently lower in older age groups than in younger age groups. Spring-levered pedometers are known to undercount steps related to obesity, so the values in this data base reflect that potential threat to validity [62]. However, their use does not completely misrepresent the general findings that steps/day differ significantly across BMI-defined obesity categories, even when measured by more sensitive accelerometers [63]. Once again, however, since pedometers are more likely to be used in clinical and public health applications, the presentation of pedometer-determined steps/day as detected in free-living populations, that include obese individuals, is relevant and therefore defensible.

It is important to consider whether we are asking the wrong question (at least for some health parameters): "How many steps/day are enough?" The question itself promotes a single-minded pursuit of threshold values, a presumed phenomenon that may not accurately characterize the true shape of a specific dose-response curve. Further, if such a threshold exists, it might only be readily achieved by a small and possibly already active subsample of any population. Recently, there has been growing interest in the study of sedentary behaviour and its potentially deleterious effects on health [64,65]. Considering this, it may be that the more appropriate question to ask in terms of pedometer-determined physical activity cut points is "How many steps/day are too few?" In support of this notion, many of the studies herein could be re-interpreted to conclude what levels of step-defined physical activity were associated with compromised health outcomes. For example, Schmidt et al. [30] reported that individuals taking < 5,000 steps/day had a substantially higher prevalence of a number of adverse cardiometabolic risk factors than those taking higher steps/day. From a public health practice point of view it is both rational and appealing to focus on motivating behaviour change in the larger portions of the population with low to very low physical activity levels rather than to focus solely on tailoring messages that may very well only appeal to subsamples that are already comparatively active. The adoption and use of a fully expanded steps/day scale that incorporates step-based translations of recommended amounts of MVPA would facilitate efforts designed to communicate both "How many steps/day are enough?" and also "How many steps/day are too few?"

In summary, it may be that specific thresholds of step-defined physical activity are associated in different ways with specific health outcomes. For example, relatively greater benefits in body composition parameters may be realized with small increments (e.g., adding 2,000 steps/day) over low levels of habitual activity in individuals who already have excess body fat, but "normalization" (with no further needed improvements) may require optimally higher physical activity levels (e.g., 11,000 to 12,000 steps/day in men, 8,000 to 12,000 step/day in women) and be relatively more difficult to achieve. Other health parameters may exhibit a more classic threshold effect, for example, positive effects on depression at ≥ 7,500 steps/day [59]. The concept of distinctly different dose-response curves related to physical activity is in keeping with the findings presented at the historic dose-response symposium in 2001 [66].

## Discussion

Human movement is not limited to bipedal locomotion, however, such locomotion is a fundamental part of daily life and is a prominent focus of public health physical activity guidelines. Steps can be accumulated throughout the day during chores, occupational requirements, child care, errands, and transportation. Walking for exercise remains the most frequently reported leisure-time activity [67]. Other types of sport and exercise can also be viewed as strategies to increase steps/day [68], but some activities, for example, swimming, and bicycling, are alternative healthy physical activities that do not easily lend themselves to tracking with pedometers [69]. We acknowledge that step-based recommendations for physical activity might be more appropriate and better received by the large segment of the population who do not regularly engage in any sport or other exercise apart from walking. Incorporating at least 30 minutes, or approximately 3,000-4,000 steps, of brisk walking should be emphasized with the promotion of any step-based recommendation, in line with public health guidelines' focus on time in MVPA. The additional benefits of engaging in even more vigorous intensity activities, and activities that do not necessarily focus exclusively on bipedal locomotion, should also be acknowledged [1].

Current public health physical activity guidelines are derived from accumulated knowledge gained over the past several decades primarily from epidemiological and intervention studies of self-reported physical activity. To be clear, messages to perform at least 30 minutes of moderate intensity activity on most, preferably all days of the week [70] (or more recently, at least 150 minutes/week in moderate intensity, 75 minutes/week in vigorous intensity physical activity, or a combination of both [1,7]) can be, for the most part, traced back to research participants' subjective descriptions of this duration, intensity, and frequency of leisure-time physical activity behaviour. The well-designed dose-response to exercise in women (DREW) study clearly demonstrated that previously sedentary women who performed even 50% of physical activity guidelines, expressed in terms of energy expenditure and objectively verified, reaped benefits in terms of significant improvements in measured cardiorespiratory fitness [56], for example. However, with the advent of body worn objective monitoring technologies there has been a keen interest in providing an objectively determined translation of the physical activity guidelines as stated, particularly with reference to time in MVPA. It is quite easy to ask someone to walk on a treadmill for 30 minutes at moderate intensity and produce a precise estimate of directly observed steps taken, for example. However, it is important to emphasize that the rich collection of research that has informed public health guidelines to date is based, for the most part, on self-reported behavior, that is, people's unique perceptions and accounts of their own behaviour. We have come to accept that, although there is a correlation [2], there is a disconnect between self-reported and objectively monitored physical activity; agreement between cross-tabulated NHANES accelerometer and self-reported physical activity data was only 18.3% (men, 20-59 y) to 32.7% (women, 60+ y) [55]. Further, those with absolutely no accelerometer-determined time spent in MVPA self-reported accumulating 43.1 to 65.2 minutes/day in MVPA [55]. To be very clear, it remains possible that self-reported frequency and time spent in absolutely defined MVPA actually equates to a lesser amount of objectively monitored behaviour than a direct and objective measurement of free-living activity, that includes the same amount of MVPA, would suggest. Alternatively, it is plausible that people have been systematically over-reporting absolute intensity of activity, as evident from the observed discrepancy between concurrent estimates of self-reported and objectively measured activity [55].

Any time a cut point of any type is set, there is an inevitable trade-off between sensitivity and specificity. Sensitivity is the proportion of true positives (values that are classified correctly as positive) relative to the sum of both true positives and false negatives and specificity is the proportion of true negatives (values that are classified correctly as negative) relative to the sum of both true negatives and false positives. For example, and hypothetically, if we set 10,000 steps/day as a cut point indicative of attaining public health guidelines that includes meeting minimal requirements for MVPA, we would anticipate that there will be some people who take 10,000 steps/day and do not accumulate 30 minutes of MVPA in at least 10-minute bouts (false positives) and also people who take less than 10,000 steps/day and still manage to accumulate 30 minutes of MPVA in at least 10-minute bouts (false negatives). This phenomenon is known [41,71-74]. If we raise the cut point to say, 12,500 steps/day, we can increase specificity and reduce the number of false positives. The trade-off is decreased sensitivity: we misclassify those who achieve sufficient MVPA at lower steps/day values. A higher cut point is desirable in research if we really want to save resources, and are willing to 'let some slip by' in a focused effort to locate those for our research studies who are most likely to be accumulating appropriate amounts of time spent in MVPA. Alternatively, if we lower the cut point to say, 7,500 steps/day, sensitivity is increased (i.e., more people meeting MVPA guidelines will be correctly classified) at the expense of decreased specificity (i.e., more people who do not meet MVPA guidelines will be incorrectly classified as if they have met them). This latter scenario is likely to be more acceptable in terms of public health strategies to communicate healthful levels of physical activity, especially if they are communicated as minimal cut points, above which additional benefits may be reaped. Regardless, it is important to realize that, whatever threshold is selected, there will be "exceptions to the rule" and these must be tolerated, otherwise confidence in any guideline can deteriorate.

Using a graduated step index as originally developed [11] to categorize escalating levels of pedometer-determined physical activity represents an important evolution beyond single value estimates of "How many steps/day are enough?" (e.g., 10,000 steps/day). Any single value, although attractive in terms of simplistic messaging, is vulnerable to "exceptions to the rule" and must be repeatedly declared with several caveats. Further, it can undermine credibility in communicating the importance of a physically active lifestyle to health at any age when it is perceived that disagreement and confusion exist. In contrast, a graduated step index has the potential to bridge research and practice because it has utility in research (e.g., reporting health outcomes across step-defined physical activity levels, tracking population levels of achievement, etc.), clinical practice (screening, prescription, compliance, etc.), behaviour change (goal-setting, self-monitoring, feedback, etc.), and public health practice (surveillance, evaluation, communication, etc.). Increased physical activity can be captured individually or on a population level by attainment of relatively higher levels within the graduated step index. The graduated levels are congruent with the now accepted concept that some activity is better than none, that increased levels of activity should be approached progressively, and that health may be optimized at higher levels, although some relatively important health benefits may be realized even with improvements over the lowest levels [1].

**Figure 1:**
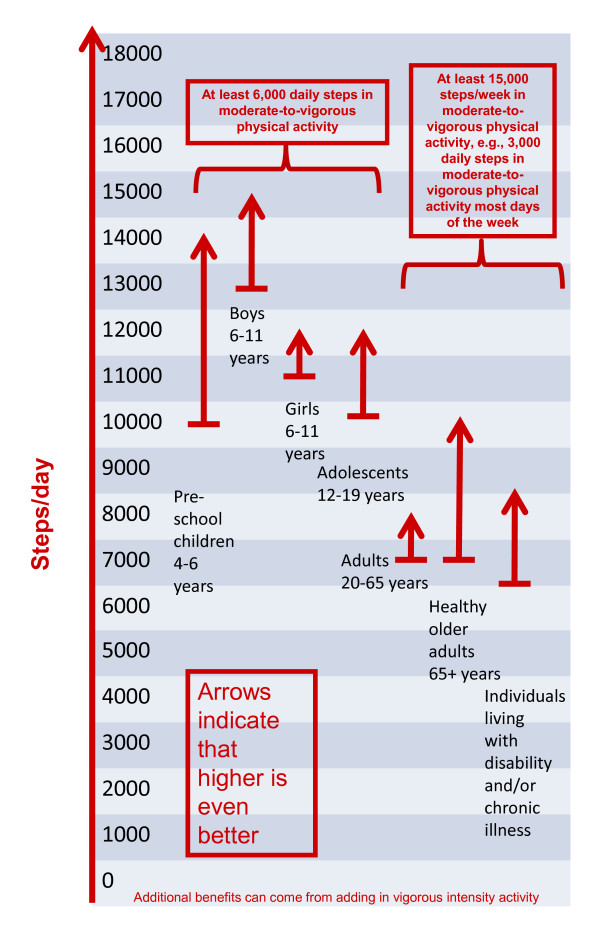
**Steps/day scale schematic linked to time spent in MVPA**.

A further improvement to the original graduated step index would be to offer a more fully expanded steps/day scale with additional "rungs on the ladder," which may be very important when applied to low active individuals and populations. Such a scale would incorporate step-based translations of public health recommendations for MVPA (e.g., superimposed on the scale), but also provide additional incremental "rungs" corresponding with roughly 10-minute bouts of activity, beginning at zero and continuing to 18,000+ steps/day, the highest mean value reported for a sample at this time: Amish men [18]. This concept is shown in Figure 1. The arrows, which suggest that more is even better, are superimposed over the fully expanded scale in Figure 1 and summarize steps/day ranges congruent with recommendations for time in MVPA across the lifespan. The base of the arrow indicates a minimal amount of recommended steps for a subgroup. For example, the range for adults is 7,000-8,000 steps/day, at least 3,000 of which should be accumulated at a brisk pace. To emphasize, this is only a threshold and the arrow indicates that more is even better. Individual and population values could be tracked and defined across the lifespan using such a common steps/day scale. Populations could be stratified and motivated and/or tracked to achieve a step/day increment coinciding with public health guidelines (e.g., 3,000 steps/day at minimally moderate intensity, and if at all possible, vigorous intensity). Smaller increments (e.g., 1,000 steps, equivalent to 10-minute bouts) could also be used to track progress on either the individual or population level. Further, as evidence continues to emerge, the likelihood of achievement of different health-related outcomes could be indicated along the graduated continuum.

A number of limitations must be acknowledged. Waist-worn pedometers and accelerometers are most sensitive to vertical accelerations (i.e., up and down motions) of the hip while ambulating (i.e., walking, jogging, running, skipping, hopping, dancing, etc.). Different devices will have different measurement mechanisms, for example, coil springs, hair springs, piezo-electric ceramics, etc., and these are patent-protected making direct comparisons between similarly named outputs challenging [75]. Differences in instrument sensitivity will affect the number of steps detected, with the greatest discrepancies resulting from divergent detection of low force accelerations. Further, as commercial items, new instrument versions appear regularly and obsolescence of specific models is always a threat [75]. However, the consistent use of research-quality pedometers does permit an opportunity for reasonable comparisons to be made across studies and between populations [28]. The instruments determined to be most suitable for the assessment of free-living physical activity have been scrutinized and include the Kenz Lifecorder, the Yamax, and the NewLifestyles NL pedometers [76]. As can be seen from the assembled tables, these instruments and other comparable instruments are well represented in research studies conducted to date. It has been noted, however, that the use of piezo-pedometers (e.g., NL series) may be more appropriate than spring-levered instruments for use in obese individuals [62]. Finally, we acknowledge that different technologies, investigators, populations, cut points, criterion measures, methodologies, etc., make rendering a simple message challenging.

## Conclusions

In summary, at least in terms of normative data, it appears that healthy adults can take anywhere between approximately 4,000 and 18,000 steps/day, and that 10,000 steps/day is a reasonable target for healthy adults, although there are notable "low active populations," including the U.S. populace [3,23]. The results of controlled studies of treadmill and over-ground walking demonstrate that there is a strong relationship between cadence and intensity, at least between 64-170 steps/minute (i.e., the values catalogued in Table 4). These cadence values can be used to generate step-based translations of minimal amounts of time in MVPA, but apply most directly to bipedal locomotor activities that produce steps. At this time the five studies [38-40,43,44] that specifically queried the number of steps in moderate intensity activity have come to similar conclusions: 100 steps/minute represents a reasonable floor value (i.e., 3 METs) that can be useful as a public health heuristic value indicative of moderate intensity walking. Multiplying this cadence by 30 minutes produces a minimum of 3,000 steps. It is important that the precision of any estimate not be overstated, but instead serve as guiding value, rather than a prescriptive one. However, an appropriate translation of physical activity guidelines, specifically allowing for minimal amounts of time in MVPA, implies that steps should be taken *over and above *those taken in the course of habitual and incidental daily activities, and also should be taken in bouts of at least 10-minutes in duration. Computed translations of free-living physical activity that also includes recommended MVPA are equivalent to 7,100 to 11,000 steps/day. Direct estimates of minimal amounts of MVPA detected in the context of monitored free-living behaviour are 7,000-8,000 steps/day. Although more weight should be given to the direct estimates, the fact that the minimal values for both are similar provides more confidence in concluding that approximately 7,000-8,000 steps/day is a reasonable threshold of free-living physical activity that is also associated with current public health guidelines' emphasis on minimal amounts of time spent in MVPA. Other levels of step-defined physical activity might be associated with various health outcomes, in keeping with current understanding of dose-response relationships. A fully expanded steps/day scale that spans a wide range of incremental increases in steps/day yet communicates step-based translations of recommended minimal amounts of time in MVPA may be useful in research and practice. Finally, regardless of the specified number of steps/day, effective programs, informed by the best research on critical moderators and mediators of behaviour change (i.e., what works best for whom under what conditions and at what cost) remain implicitly necessary in terms of increasing individual and population levels of ambulatory activity.

## Competing interests

The following authors declare they have no competing interests: CT-L, WJB, SAC, KDC, BG-C, YH, SI, SMM, NM, J-M O, DAR, MDS, GMS, JCS, PJT, and MAT. CLC is associated with the Canadian Fitness and Lifestyle Research Institute which is funding in part by the Public Health Agency of Canada (PHAC). SNB receives book royalties (<$5,000/year) from Human Kinetics; honoraria for service on the Scientific/Medical Advisory Boards for Alere, Technogym, Santech, and Jenny Craig; and honoraria for lectures and consultations from scientific, educational, and lay groups. During the past 5-year period SNB has received research grants from the National Institutes of Health, Department of Defence, Body Media, and Coca Cola.

## Authors' contributions

CT-L and CLC conceived and designed the project. CT-L acquired the data and prepared analysis for initial interpretation. All authors contributed to subsequent analysis and interpretation of data. CT-L prepared a draft of the manuscript. All authors contributed to critically revising the manuscript for important intellectual content. MAT, SAC, and DAR edit checked the tables. All authors gave final approval of the version to be published and take public responsibility for its content.
